# LDR-Net: Landmark-Guided Diverse Regional Representation Learning for Facial Expression Recognition

**DOI:** 10.3390/biomimetics11070503

**Published:** 2026-07-17

**Authors:** Yansha Lu, Faliang Chang, Chunsheng Liu, Hui Liu, Yiming Huang

**Affiliations:** School of Control Science and Engineering, Shandong University, Jinan 250061, China; yanshalu1016@mail.sdu.edu.cn (Y.L.); huiliu@mail.sdu.edu.cn (H.L.); 202014769@mail.sdu.edu.cn (Y.H.)

**Keywords:** facial expression recognition, human visual system, landmark-guided representation, diverse regional representation, occlusion-robust and pose-invariant

## Abstract

Reliable facial expression recognition (FER) is essential for human–computer interaction across scenarios. However, existing methods often overlook spatial structure when seeking region-specific features due to fixed or limited local regions, limiting representation capability under real-world variations. Inspired by the multi-location attention of the human visual system, we propose a Landmark-guided Diverse Regional Representation Network (LDR-Net), using dynamic landmarks as patch centers to preserve structural details while locating expression-critical areas, facilitating effective regional representations for FER. First, a novel Diverse Regional Feature Extraction (DRFE) module operates via complementary operations: landmark-guided cropping for local details and cross-level integration with feature reorganization for holistic aggregation. Second, a novel Diverse Representation Learning (DRL) module is proposed with a collaborative dual-stream mechanism that captures fine-grained local dependencies via Transformers while reinforcing global features through attention-based enhancement, enabling comprehensive feature learning. Finally, a new Hybrid Feature Fusion (HFF) module is proposed for joint decision optimization via a hierarchical hybrid strategy, which aggregates intra-branch predictions followed by weighted branch-level fusion. Experiments on three FER benchmarks (RAF-DB, AffectNet, SFEW) and five occlusion/pose test sets demonstrate that LDR-Net outperforms state-of-the-art methods, while cross-scene validation on KMU-FED confirms its effectiveness in real-world driving.

## 1. Introduction

Facial expressions are a direct reflection of human emotions and play a crucial role in communication. Automatic facial expression recognition (FER) has emerged as a key emotion analysis tool in artificial intelligence (AI), with applications in human–computer interaction, medical diagnosis, driver status monitoring, and online education [1]. These applications enhance user experience and ensure safety, highlighting the importance of FER across diverse fields.

**Figure 1:**
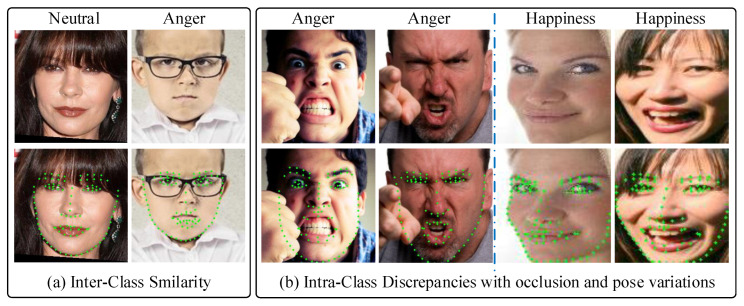
Key challenges and facial landmark invariance in FER. Panel (**a**) illustrates inter-class similarity across different categories, while panel (**b**) shows intra-class discrepancies caused by occlusions and pose variations. Notably, facial landmarks serve as a sparse representation of key facial regions, exhibiting remarkable invariance to these real-world variations, suggesting a potential anchor for robust feature extraction. The images are sourced from RAF-DB [2] and FED-RO [3].

Unlike general image classification, FER must handle facial variations, which present notable inter-class similarities and intra-class discrepancies. In recent years, FER has achieved excellent performance on laboratory-controlled datasets, which typically feature uniform illumination, frontal pose, and no occlusion. However, FER in the wild usually suffers from difficulties including occlusion, pose variation, illumination variation and insufficient quality. These real-world variations impose higher demands on representation capability, making FER in the wild a challenging task, as illustrated in Figure 1.

To address the challenges of FER in the wild, existing approaches can be broadly categorized into global-based, local-based, global–local fusion, and facial landmark-based methods. Global-based methods focus on extracting holistic facial features, capturing overall structural and semantic information [4]. Local-based methods, such as patch-based strategies [5] and relation-aware learning [6], focus on fine-grained spatial information. Global–local fusion methods integrate both scales to improve robustness against occlusion and pose variation [7,8]. Additionally, facial landmark-based methods enhance feature extraction by focusing on expression-relevant regions [3,9,10]. Among these, combining global features with patch-based local feature design has emerged as a mainstream direction to address real-world challenges. However, the local patch extraction in these methods often relies on fixed or limited local regions (e.g., uniformly divided grids or hand-crafted patches). This can lead to two limitations: spatial structure tends to be overlooked when seeking region-specific features, and the network may struggle to dynamically adapt to expression-relevant regions that vary across faces and scenarios. Consequently, their representations may be constrained under real-world variations. Facial landmarks provide stable structural cues relatively robust to pose and occlusion variations, and have been widely adopted in FER. Existing methods typically employ landmarks for alignment, region selection [11], geometric modeling [12], or auxiliary guidance [13], yet often in a fixed or indirect manner without fully integrating them into representation learning. In contrast, we treat landmarks as dynamic spatial anchors to guide adaptive region generation, enabling structure-aware and flexible feature learning under real-world variations.

Inspired by multi-location attention of the human visual system, we propose the Landmark-guided Diverse Regional Representation Network (LDR-Net), which leverages facial landmarks as dynamic spatial anchors to localize expression-critical regions while preserving structural details, promoting efficient region-specific representations for robust FER. The network can be interpreted with three bio-inspired principles: (1) using facial landmarks as explicit spatial anchors to mimic the human visual system (HVS)’s salient feature selection, (2) learning diverse regional representations in parallel to emulate the HVS’s distributed attention, and (3) integrating local and global features to replicate the HVS’s holistic perception. Specifically, the Diverse Regional Feature Extraction (DRFE) module extracts diverse regional representations via complementary operations: landmark-guided cropping dynamically crops patches anchored at facial landmarks for local details, while cross-level integration with feature reorganization aggregates multi-level features for global context, establishing complementary local–global representations while preserving structural details. Second, the Diverse Representation Learning (DRL) module employs a complementary dual-stream mechanism: Transformer-based Local Relation-aware Representation (LRR) cells capture fine-grained local dependencies among landmark-centered patches, while CNN-based Global Reinforced Representation (GRR) cells enhance global features through attention-based reinforcement. This enables comprehensive feature learning that integrates local relational cues with holistic facial representations. Finally, a Hybrid Feature Fusion (HFF) module is introduced for joint decision optimization via a hierarchical hybrid strategy that aggregates intra-branch predictions and performs weighted branch-level fusion, balancing local and global features for robust classification.

Our main contributions are summarized as follows.

- We propose a novel bio-inspired Landmark-guided Diverse Regional Representation Network (LDR-Net) that moves beyond fixed or limited local regions by using facial landmarks as dynamic patch centers to locate expression-critical areas while preserving structural details, facilitating diverse regional representations for robust FER.
- We develop a Diverse Regional Feature Extraction (DRFE) module that captures local details via landmark-guided cropping and aggregates multi-level features via cross-level integration, enabling both fine-grained local details and holistic facial reinforcement.
- We propose a Diverse Representation Learning (DRL) module with a complementary dual-stream mechanism: Transformer-based Local Relation-aware Representation (LRR) cells capture fine-grained local dependencies among landmark-centered patches, while CNN-based Global Reinforced Representation (GRR) cells reinforce global features through attention-based enhancement, enabling comprehensive feature learning.
- Our LDR-Net substantially boosts FER performance under challenging conditions, achieving state-of-the-art accuracies of 92.11% on RAF-DB, 67.14% on AffectNet-7, 63.20% on AffectNet-8, and 62.65% on the SFEW dataset. Additionally, it achieves near-perfect accuracy (99.10%) on the KMU-FED driver facial expression dataset, showcasing its real-world effectiveness.

## 2. Related Work

### 2.1. Facial Expression Recognition

FER enables machines to understand human emotions and make judgments based on psychological states. Early FER was predominantly based on datasets collected in lab-controlled environments. With the growing demand for technological solutions to real-world challenges, there is a gradual shift towards large-scale data acquisition in the wild. According to the type of features, FER methods can be broadly categorized into traditional hand-crafted methods and deep learning-based methods.

Traditional methods mainly include texture-based, geometry-based, and hybrid approaches. Texture-based methods rely on hand-crafted features such as histograms of oriented gradients (HOGs) [14], local binary patterns (LBPs) [15], and scale-invariant feature transform (SIFT) [16]. These methods are limited by manual annotation and low generalization ability. Geometry-based methods leverage geometric patterns of facial shape, such as coordinate information of pre-defined facial landmarks [17] or geometric features like distances and angles between landmarks [18]. Hybrid feature extraction methods combine two or more traditional approaches to further enrich feature representation.

### 2.2. Efficient FER with Representation Reinforcement

Deep learning can automatically extract spatial features of images without manual intervention [19]. In recent years, deep learning methods have made breakthrough progress in FER tasks [20], especially on challenging large-scale datasets. To achieve efficient and robust FER, existing deep learning methods primarily focus on enhancing representational ability [7], designing novel loss functions [21], and reducing data uncertainties [20,22].

To improve representational ability via network design and integration, Ruan et al. [4] propose FDRL to capture expression-specific variations from a global perspective. Wang et al. [1] propose RAN to encourage high attention weights for the most discriminative regions. From a global–local combination perspective, Zhao et al. [7] propose MA-Net to learn deep global multi-scale and local attention features. Among integrated approaches, Wang et al. [23] propose MGR^3^Net, which reinforces representation via attention-guided global–local fusion, while Tao et al. [24] introduce a hierarchical attention network with progressive feature fusion. Recently, Dong et al. [25] propose WGGLFA, which leverages wavelet-guided global–local feature aggregation for robust FER. More recently, MHAN [26] introduces a multi-head hybrid attention mechanism to capture fine-grained regional dependencies, further enhancing discriminative feature learning. These advances validate the benefits of strengthened feature interactions, motivating further exploration of representation learning with explicit spatial encoding.

### 2.3. Efficient FER Based on Facial Landmarks

Facial landmarks provide compact geometric representations of facial structures and have been widely adopted in FER. Early works exploit hand-crafted geometric features, such as distances and angles between pre-defined landmarks [17,18], which are computationally efficient but have limited representational capacity.

With the development of deep learning, facial landmarks are often used as structural priors or auxiliary supervision signals to enhance feature learning. Representative works incorporate landmarks for joint facial representation learning [11] or use them as guidance under different learning frameworks [13,27,28]. Another line of research models landmarks as graph structures, where GNNs are applied to capture spatial dependencies among facial components [12]. Hybrid and multi-task frameworks have also been explored in landmark-related facial analysis. For instance, MOL [29] jointly models micro-expression recognition, optical flow, and landmark prediction, demonstrating the potential of multi-task learning with landmark information, though it focuses on micro-expressions.

Despite these advances, most existing methods still rely on fixed structural modeling or indirect landmark supervision, which limits flexible region-level reasoning. This motivates our adaptive landmark-based representation learning with explicit spatial reasoning beyond fixed structural modeling.

### 2.4. Efficient FER with Vision Transformer

Vision Transformer is a transduction model that relies entirely on self-attention to compute input and output representations. Initially introduced for NLP tasks, Vision Transformer has recently been widely adopted in computer vision due to its excellent ability to model long-range dependencies [30].

In FER tasks, Xue et al. [6] first propose a Transformer-based method named TransFER, demonstrating the importance of relations among local patches. Xue et al. [31] introduce a Vision Transformer with Attentive Pooling (AP) modules to focus on discriminative features and reduce the impact of noisy ones. Kim et al. [32] propose Squeeze ViT to combine global and local features for FER. Cheng et al. [33] propose MRAN to capture efficient emotional features by exploring relationships between global and local attention features. Liang et al. [34] propose a two-branch network that leverages a Vision Transformer for global feature extraction and a CNN for local feature learning, integrating both through adaptive weighting to enhance FER accuracy in the wild.

Despite these advances, most existing methods, whether landmark-based or Transformer-based, rely on fixed keypoint localization or fixed patch partitioning, which often disrupts spatial structure and overlooks spatial coherence. Motivated by these observations, we introduce inherent relation learning with dynamic patch anchoring to preserve spatial structure while capturing diverse region-specific features.

## 3. Proposed Method

### 3.1. Overview

**Figure 2:**
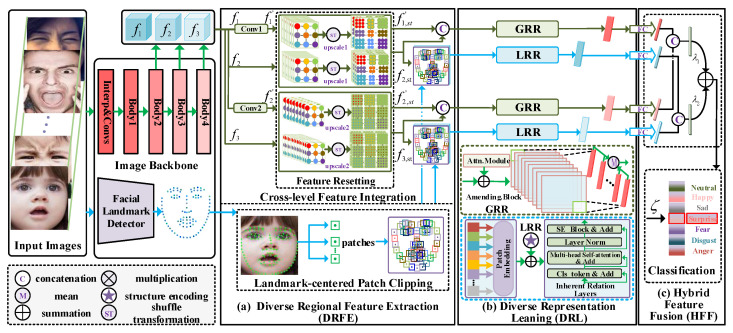
The structure of the proposed LDR-Net. Initially, we extract multi-level features and identify facial landmarks. (**a**) DRFE extracts diverse regional features via landmark-centered patch clipping, feature resetting, and cross-level feature integration. (**b**) DRL captures fine-grained local dependencies via Local Relation-aware Representation (LRR) cells and reinforces global features via Global Reinforced Representation (GRR) cells. (**c**) HFF performs joint decision optimization via intra-branch aggregation and weighted branch-level fusion. Different colors are used solely to distinguish different functional modules for visualization purposes. The ellipsis in the input images denotes multiple representative facial images rather than omitted network components. The red frame indicates the expression category (‘Surprise’) corresponding to the illustrated input facial image.

The proposed Landmark-guided Diverse Regional Representation Network (LDR-Net) consists of three main components, as shown in Figure 2. Given face images, a backbone is utilized to extract reliable multi-level features, while a facial landmark detector is employed to identify accurate facial landmarks. Firstly, Diverse Regional Feature Extraction (DRFE) is proposed to extract efficient region-specific representations while preserving structural details, by clipping landmark-centered regions, resetting features, and integrating cross-level information, as shown in Figure 2a. Then, Diverse Representation Learning (DRL) is proposed to learn diverse regional representations with Local Relation-aware Representation cells (LRR) and Global Reinforced Representation cells (GRR), as shown in Figure 2b. Finally, Hybrid Feature Fusion (HFF) performs joint decision optimization via a hierarchical hybrid strategy that aggregates intra-branch predictions followed by weighted branch-level fusion for the final FER classification, as shown in Figure 2c.

### 3.2. Diverse Regional Feature Extraction (DRFE) Module

The DRFE module extracts diverse regional representations while preserving structural details via two complementary operations: landmark-guided cropping for local details and cross-level integration with feature reorganization for global context, thereby laying the foundation for subsequent modeling of relationships between local regions and the aggregated global representations. Specifically, we adopt IR50 [35] as the backbone and extract its last three hierarchical feature maps. For local details, landmark-centered patch clipping is applied to the middle and deep layers, yielding two local outputs. For global context, feature resetting and cross-level feature concatenation are applied to all three layers, producing two global outputs. A facial landmark detector (e.g., SLPT [36]) provides dynamic patch centers for the local stream. The following subsections detail these operations.

**Landmark-centered Patch Clipping**: Leveraging facial landmarks as dynamic patch centers, landmark-centered patch clipping effectively focuses on critical facial regions and enhances the contextual relevance of local feature embedding.

As stated by Dosovitskiy et al. in [37], ViT can divide an image or a feature map $I\in R^{H_{I}\times W_{I}\times C}$ into a grid of $\frac{H_{I}}{P_{h}}\times \frac{W_{I}}{P_{w}}$, with each patch having a size of $P_{h}\times P_{w}$, and map it into a *d*-dimension vector as input. Unlike ViT, landmark-centered patch clipping crops a local patch for each facial landmark, positioning the patch center at the landmark itself. Each patch is clipped with the fixed size ($P_{h},P_{w}$) from the feature map. Then, the landmark-centered patches are resized to K×K and mapped into a series of vectors through a CNN block with multiple convolutional layers. Each vector can be viewed as the representation of the corresponding landmark-centered patch. Specifically, we extract N=98 facial landmarks using the detector from SLPT [36] to serve as patch centers. This processing enables the learning of the inherent relations among these local vectors, facilitating the efficient capture of multiple prominent expression-specific features.

**Feature Resetting**: The multi-level features are reset as groups of large-scale feature representations via shuffle transformation (i.e., PixelShuffle). This operation clusters peripheral features of the same erosion degree together, promoting mutual complementarity among different-level sub-pixel features. It changes only the absolute positions of feature points while preserving their relative positions, thus improving representation learning efficiency by reducing convolution operations and mitigating the erosion effect. The process can be formalized as follows.

Suppose the input image $X\in R^{H\times W\times C}$ is a low-resolution image, and the upscale factor *r* is a magnification factor. The shuffle transformation can upsample the input image to a high-resolution image $Y\in R^{Hr\times Wr\times C/r^{2}}$. Here, $F_{st,r}$ denotes the operation of PixelShuffle with upscale factor *r*, which can be expressed as follows:

$$Y=F_{st,r}\left(X\right),$$

The coordinate mapping is given by the following:

(1) $$Y_{i,j,c^{\prime }}=X_{\left\lfloor i/r\right\rfloor ,\;\left\lfloor j/r\right\rfloor ,\;c},\;\mathrm{with}\;c=c^{\prime }\cdot r^{2}+\left(i\;\mathrm{mod}\;r\right)\cdot r+\left(j\;\mathrm{mod}\;r\right),\;$$

where 0≤i<Hr, 0≤j<Wr, $0\leq c^{\prime }<C/r^{2}$, 0≤c<C, and *C* is divisible by $r^{2}$.

**Cross-level Feature Integration**: A process that integrates features from different hierarchical levels into the current layer through convolutional and concatenation operations. This operation aims to enrich the diversity of features and mitigate the limitations of the receptive field. Together with feature resetting, it transforms the multi-level backbone features into two globally aggregated outputs, providing rich and complementary representations for subsequent learning.

### 3.3. Diverse Representation Learning (DRL) Module

The DRL module learns diverse regional features through a collaborative dual-stream mechanism. It employs Transformer-based Local Relation-aware Representation (LRR) cells to capture fine-grained dependencies among landmark-centered patches, while CNN-based Global Reinforced Representation (GRR) cells enhance global features through attention-based reinforcement, ensuring comprehensive facial representation learning.

#### 3.3.1. Local Relation-Aware Representation Cells (LRR)

LRR leverages the Transformer’s strength in relational modeling. It comprises three components: Patch Embedding (PE), Structure Embedding (SE), and Inherent Relation Layers, which together capture relationships among landmark-centered patch features and guide attention to salient regions.

**Patch Embedding (PE)**: In the proposed PE, each landmark-centered patch is resized to K×K and mapped into a vector through a CNN block with multiple convolutional layers. Each vector serves as the representation of the corresponding patch.

**Structure Embedding (SE)**: The SE is represented by a series of learnable parameters, which can retain the relative position information of facial landmarks in regular face shape.

**Inherent Relation Layers**: The Inherent Relation Layers are designed to model the relations among features from landmark-centered patches. They are composed of Cls_token, Multi-head Self-attention, Layer Norm, and SE_Block.

Specifically, Multi-head Self-attention architecture consists of a stack of blocks; each of them includes a LayerNorm, Attention (Multi-head Self-attention), Dropout, an additional LayerNorm, and an MLP. Based on the self-attention mechanism in the Inherent Relation Layers, we can learn the inherent relation between tokens in an adaptive interactive manner.

Given the input image $I\in R^{H_{I}\times W_{I}\times C}$, the landmark-centered patch features $X_{\mathrm{lm}}\in R^{N\times D}$ are mapped into three matrices: landmark query matrix $Q_{\mathrm{lm}}$, landmark key matrix $K_{\mathrm{lm}}$, and landmark value matrix $V_{\mathrm{lm}}$:

(2) $$Q_{\mathrm{lm}}=X_{\mathrm{lm}}W^{q},\;K_{\mathrm{lm}}=X_{\mathrm{lm}}W^{k},\;V_{\mathrm{lm}}=X_{\mathrm{lm}}W^{v},\;$$

where $W^{q},W^{k},W^{v}\in R^{D\times D}$ are the projection matrices. The attention operation is defined as follows:

(3) $$\mathrm{Attention}\left(Q_{\mathrm{lm}},K_{\mathrm{lm}},V_{\mathrm{lm}}\right)=\mathrm{Softmax}\left(\frac{Q_{\mathrm{lm}}K_{\mathrm{lm}}^{T}}{\sqrt{d_{k}}}\right)V_{\mathrm{lm}},\;$$

where Softmax(·) is the softmax activation function and $1/\sqrt{d_{k}}$ is the scaling factor. The encoder output $X_{lm,o}$ is then obtained as follows:

$$X_{\mathrm{lm}}^{\prime }=\mathrm{Attention}\left(Q_{\mathrm{lm}},K_{\mathrm{lm}},V_{\mathrm{lm}}\right)+X_{\mathrm{lm}},$$

(4) $$X_{\mathrm{lm},o}=\mathrm{MLP}\left(\mathrm{Norm}\left(X_{\mathrm{lm}}^{\prime }\right)\right)+X_{\mathrm{lm}}^{\prime },\;$$

where MLP(·) is a multi-layer perceptron and Norm(·) denotes layer normalization.

#### 3.3.2. Global Reinforced Representation Cells (GRR)

GRR leverages the advantages of CNNs in capturing rich global semantic information while mitigating their limitations, thereby reinforcing the learning of global representations. GRR mainly consists of Attn.Module and Amend.Block.

**Attn.Module**: When the cross-level feature integration is completed, an Attn.Module is constructed based on Channel Attention Module (CAM) to implement the global AdaptiveAvgPool and AdaptiveMaxPool, assigning the weights of different channels. When the reset global features are set to *f*, the output of Attn.Module, $f_{AM}$, is represented as follows,

(5) $$f_{AM}=F_{fc}\left(F_{Avg}\left(f\right)\right)+F_{fc}\left(F_{Max}\left(f\right)\right)+f,\;$$

where $F_{Avg}$ and $F_{Max}$ denote the operation of AdaptiveAvgPool and AdaptiveMaxPool, and $F_{fc}$ is two fully connected layers.

**Amending.Block**: Subsequently, an Amending.Block is constructed by a special convolutional layer, which can dilute the weight of the peripheral pixels, along with a batch normalization layer. Considering the characteristic distribution of erosion information in the reset feature maps, the special convolutional layer should be characterized by no padding, large convolution kernel, and big stride. In this way, we can ignore more erosion information from different receptive fields and weaken the adverse effects of convolution operations.

### 3.4. Hybrid Feature Fusion (HFF) Module

We propose a Hybrid Feature Fusion (HFF) module for joint decision optimization via a hierarchical hybrid strategy. As illustrated in Figure 2c, it operates in two steps: intra-branch prediction aggregation followed by weighted branch-level fusion.

#### 3.4.1. Step 1: Intra-Branch Prediction Aggregation

Given the two global outputs ($G_{1},G_{2}$) and two local outputs ($L_{1},L_{2}$) from the DRL module, we first apply a fully connected layer to each output independently. The global branch representation *G* is obtained by concatenating the results from $G_{1}$ and $G_{2}$, and similarly the local branch representation *L* from $L_{1}$ and $L_{2}$:

$$G=\mathrm{Concat}\left(\mathrm{FC}\left(G_{1}\right),\mathrm{FC}\left(G_{2}\right)\right),\;L=\mathrm{Concat}\left(\mathrm{FC}\left(L_{1}\right),\mathrm{FC}\left(L_{2}\right)\right).$$

#### 3.4.2. Step 2: Weighted Branch-Level Fusion

We then apply a fully connected layer to *G* and *L* separately to obtain the final global and local predictions, followed by weighted fusion:

(6) $$F=w_{G}\cdot \mathrm{FC}\left(G\right)+w_{L}\cdot \mathrm{FC}\left(L\right),\;$$

where $w_{G}$ and $w_{L}$ are learnable weight coefficients balancing the contributions of the global and local branches.

## 4. Experiments

To comprehensively evaluate the proposed LDR-Net, we conduct extensive experiments on public FER benchmarks, occlusion/pose test sets, and a driver facial expression dataset for real-world validation. We describe the datasets, implementation details, and evaluation metrics, followed by ablation studies on RAF-DB. Comparative analysis with several state-of-the-art (SOTA) methods further demonstrates its superiority, while cross-scene validation on KMU-FED confirms its effectiveness in real-world driving.

### 4.1. Datasets

**RAF-DB** [2] is a real-world facial expression dataset which contains 30,000 facial images downloaded from the Internet. In our experiments, only the single-labeled subset is used for evaluations. RAF-DB contains 15,339 images with seven categories, of which the number of the training set and the testing set is 12,271 and 3068, respectively. **AffectNet** [38] is the largest facial expression dataset so far, with a total of more than one million images collected from the Internet. Our experiments select manually labeled images that contain two benchmark branches, AffectNet-7 and AffectNet-8. For AffectNet-7, there are 283,901 training images and 3500 validation images with seven basic expressions. AffectNet-8 introduces an additional category of *contempt*, and expands the training and test samples to 287,568 and 4000, respectively. **SFEW** [39] is a static facial expression dataset. The most commonly used version is SFEW 2.0, which contains three sets with seven basic categories. There are 958 samples in the training set, 436 samples in the validation set and 372 samples in the test set.

**FED-RO** [3] (Facial Expression Dataset with Real-world Occlusions) is a facial expression dataset with real occlusion for FER in the wild. It contains 400 images with seven basic expressions. **Occlusion-RAF-DB and Occlusion-AffectNet** are the two subsets used to evaluate the performance of the FER model in real-world occlusion conditions. The Occlusion-RAF-DB and Occlusion-AffectNet contain 735 and 683 images, respectively. **Pose-RAF-DB and Pose-AffectNet** are the two subsets used to value the performance of the FER model under varying pose conditions. The Pose-RAF-DB selects images with a pitch or yaw angle greater than 30° and 45°, containing 1248 and 558 images, respectively. Similarly, the Pose-AffectNet selects images with a pitch or yaw angle greater than 30° and 45°, containing 1948 and 985 images, respectively.

**KMU-FED** [18] (Keimyung University Facial Expression of Drivers) consists of 1106 images from 12 subjects, each exhibiting six basic emotions: *anger*, *disgust*, *fear*, *happiness*, *sadness*, and *surprise*. The images were collected in real-world driving environments using near-infrared cameras, which were exposed to varying lighting conditions and partial occlusions.

Table 1 presents the expression category distribution for each dataset used in our experiments.

### 4.2. Implementation Details and Metrics

For all datasets, the official aligned face images are selected and then resized to 224 × 224 pixels. Random horizontal flipping and random erasing are employed to avoid over-fitting. The image backbone is IR50 [35] pre-trained on MS-Celeb-1M [40], which is designed to extract multi-level features. The initial facial landmarks are provided by a facial landmark detector (e.g., SLPT [36]), which outputs the 2D coordinates of 98 facial landmarks. All detected landmarks are utilized without additional filtering. Our method is implemented with the PyTorch (version 2.4.0) toolbox on a NVIDIA GeForce RTX 4090 platform (NVIDIA Corp., Santa Clara, CA, USA), using the Adam optimizer (PyTorch built-in) with a momentum of 0.9. The learning rate is initialized as 3.5 × 10^−4^ and a weight decay of 1 × 10^−4^ is used.

Our model contains two Vision Transformer branches, each with a depth of two Transformer layers and eight attention heads. The embedding dimensions are set to 512 and 1024, respectively. The batch size is set to 128 for RAF-DB, AffectNet, and FED-RO, and 64 for SFEW due to its smaller dataset size.

The evaluation metrics used are overall sample accuracy (Acc) and mean class accuracy (Mean Acc).

### 4.3. Ablation Studies on RAF-DB Dataset

#### 4.3.1. Analysis of Different Components in LDR-Net

To evaluate the effectiveness of each component within LDR-Net, the ablation studies are conducted on the RAF-DB [2] dataset. The study primarily involves DRFE, DRL, HFF, additional global streams of DRFE (DRFE_Gs), and global streams of DRL (GRR). Here, we investigate the contributions of DRFE and DRL in a branching manner, i.e., evaluating the global and local branches separately.

The experimental results of different variants of our model are presented in Table 2. Set (a) shows the evaluated Acc and Mean Acc for our baseline model (IR50 [35]). Compared with the baseline, incorporating DRFE_Gs results in a performance improvement of 0.72% and 0.56% in Set (b), respectively. In Set (c), further adding GRR (the global streams of DRL) leads to additional improvements of 0.23% and 0.27%, respectively. This suggests the global streams of DRFE and GRR can discriminate important complementary features from different levels and adaptively improve the efficiency of global representation learning. The Set (d) incorporates a DRFE designed to capture diverse regional features, achieving improvements of 1.01% and 0.45% over the baseline model. In Set (e), adding DRL to the architecture (DRFE + DRL) further boosts performance to 91.92% accuracy and 86.14% mean accuracy. Finally, an ensemble of all components in Set (f) further enhances accuracy to 92.11% and mean accuracy to 86.55% on RAF-DB. Therefore, we conclude that each component contributes positively, and their integration (LDR-Net) delivers the optimal performance across all variants.

#### 4.3.2. Analysis of the Cross-Level Feature Integration in DRFE

**Figure 3:**
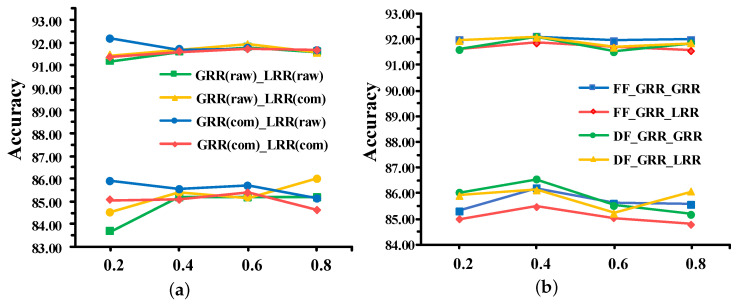
Performance comparison of different combination strategies and the varying fusion weight coefficients. (**a**) Decision-level Fusion Weight; (**b**) Diverse Fusion Weight.

To optimize the cross-level feature integration in DRFE, we incorporate integration across both global and local branches, labeling integrated branches as “com”, and non-integrated ones as “raw”. Therefore, four combinations are constructed: “GRR(raw)_LRR(raw)”, “GRR(raw)_LRR(com)”, “GRR(com)_LRR(raw)”, and “GRR(com)_LRR(com)”. We compare the Acc performance of different combinations in decision-level fusion under varying weight coefficients, as shown in Figure 3a. Comparing the trend of different combinations, it is obvious that “GRR(com)_LRR(raw)” has a notable advantage over other combinations in terms of both Acc and Mean Acc. The benefits of GRR from this integration may be attributed to the fact that “com” primarily enriches the current layer’s features by incorporating convolutional features from adjacent layers through the design of specific convolutional blocks. While LRR emphasizes local regions within the current layer, the complementary features from other levels may reduce the local feature layer’s focus on specific prominent regions.

#### 4.3.3. Analysis of Patch Size (PS) in LRR and Kernel Size (KS) in GRR

**Figure 4:**
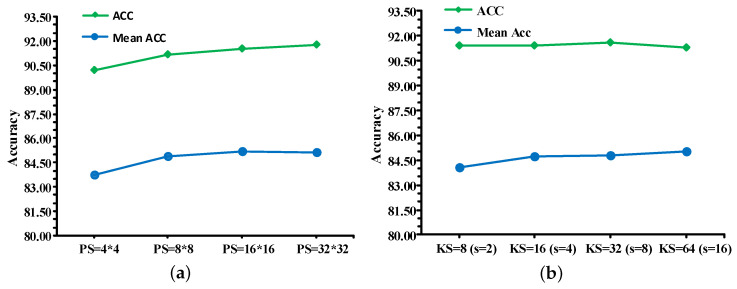
Evaluation of the patch size (PS) in LRR and the kernel size (KS) in GRR. (**a**) Patch Size; (**b**) Kernel Size (stride).

To select the optimum patch size (PS) for the Inherent Relation Layers in LRR and kernel size (KS) for the convolutional layer in the Amending.Block in GRR, we conduct experiments to set the PS as (4, 4), (8, 8), (16, 16), and (32, 32), and gradually increase the KS from $2^{3}$ to $2^{6}$ evaluated on RAF-DB, as shown in Figure 4a,b, respectively. From left to right within each patch size, the improvements in accuracy become smaller and even degrade. Considering that a large patch size requires huge computation resources, we choose (16, 16) as the optimal PS. With the increase in the number of KS, the mean Acc keeps growing as expected while Acc presents a pattern of low initial growth followed by decay. Considering that excessively large convolution kernels can significantly increase computational costs and cause the loss of detail in feature maps, we select KS = $2^{5}$ as the optimal value.

#### 4.3.4. Evaluation of Fusion Strategy in LDR-Net

We also conduct experiments to evaluate different fusion strategies, which include feature-level, decision-level, and hybrid-level fusion, as shown in Table 3. Feature-level fusion means directly concatenating all learned feature vectors into a joint feature vector, and trains a classifier for FER, while the decision-level fusion combines all the recognition results, obtained via FC layers from learned features, into a joint feature vector. The results indicate that decision-level fusion is superior to feature-level fusion. This insight prompts us to further explore ways to maximize decision-level advantages in LDR-Net.

From the results in Table 3, we observe that the hybrid-level fusion is a more suitable strategy for our proposed LDR-Net than the other strategies. Particularly noteworthy is the superiority of our hybrid-level decision strategy in mean Acc, which is 1.22% higher than that of simple decision-level strategies. The main reason is the strong coupling of holistic–regional features and the powerful synergy of different-level features for the final decision.

#### 4.3.5. Analysis of Diverse Feature Fusion Strategy in HFF

To explore the optimal feature fusion strategy in HFF, we conduct four comparative experiments with different feature fusion methods. We label feature-level fusion as "FF" and the decision-level fusion as “DF”. “GRR_GRR” refers to concatenating multi-level, same-type features to derive joint representations for the decision layer. “GRR_LRR” represents the concatenation of global–local features at the same level to generate joint representations.

The comparative results are shown in Figure 3b, with the results for different weights displayed from left to right. The comparison of performance trends under the FF fusion strategy indicates that GRR_GRR performs notably better than GRR_LRR. This observation is consistent with previous findings regarding the impact of cross-level feature integration on global and local features. Analysis of performance under different fusion strategies reveals that DF outperforms FF overall but shows a greater sensitivity to the weight coefficient. DF_GRR_GRR with λ=0.4 is selected as the optimal fusion strategy.

#### 4.3.6. Visualization of the Expression Representations with t-SNE on RAF-DB Dataset

**Figure 5:**
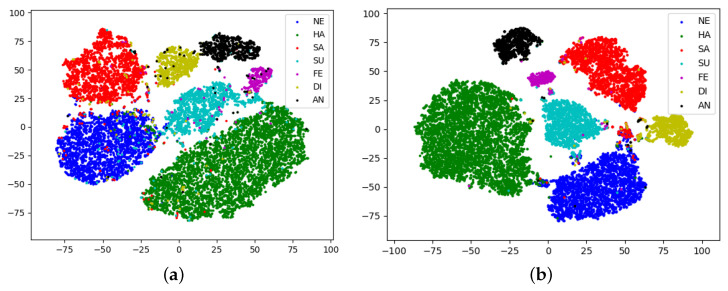
The visualization of the expression representations with t-SNE [41] for the whole RAF-DB dataset. (**a**) Baseline features; (**b**) Features learned by LDR-Net.

We use t-SNE [41] to visualize the expression representations extracted by the baseline (IR50) and the proposed LDR-Net on the 2D space, respectively, as illustrated in Figure 5. We observe that the expression representations extracted from our LDR-Net method effectively reduce inter-class similarity and intra-class discrepancy across different expressions, leading to clearer and more distinct classification boundaries. Notably, when compared to the baseline, LDR-Net makes the differences between *fear* and *surprise*, as well as *disgust* and *sadness*, more distinct.

### 4.4. Comparisons with the State-of-the-Art (SOTA) Methods

#### 4.4.1. Comparison on RAF-DB Dataset

The comparison with several SOTA methods on the RAF-DB dataset is presented in Table 4. Our full LDR-Net (with IR-50 backbone and facial landmarks) achieves 92.11% accuracy, outperforming the previous best method (MGR^3^Net [23] with ResNet-50) by 1.06%. Notably, with the same IR-50 backbone, LDR-Net surpasses TransFER [6] (90.91%) by 1.20%. The ResNet-18 variant of LDR-Net, which does not use facial landmarks, also achieves competitive performance (88.95%), suggesting that the proposed representation learning strategy is effective even without landmark guidance. These results demonstrate the superiority of LDR-Net in capturing discriminative expression features.

#### 4.4.2. Comparison on AffectNet Dataset

We also compare LDR-Net with several SOTA methods on the AffectNet-7 and AffectNet-8 datasets, as shown in Table 5. Considering the substantial class imbalance in the AffectNet dataset, we employ an oversampling strategy following RAN [1] and MA-Net [7]. On AffectNet-7, LDR-Net achieves 67.14% accuracy, outperforming the previous best method TransFER [6] (66.23%) by 0.91% and the IR-50 baseline (64.91%) by 2.23%. On AffectNet-8, LDR-Net achieves 63.20% accuracy, surpassing the baseline (61.75%) by 1.45% and EDGL-FLP [45] (61.25%) by 1.95%. These results highlight the superior ability of LDR-Net in capturing expression-specific features, further validating its effectiveness.

#### 4.4.3. Comparison on SFEW Dataset

We also compare the LDR-Net with several SOTA methods on the SFEW dataset, as shown in Table 6. A naive model fusion conducted by RAN [1] obtains 56.40%. MA-Net [7] improves its performance to 59.40% through an effective combination of global and local representations. Notably, FDRL [4] achieves a remarkable result of 62.16%, which benefits from the feature decomposition and reconstruction. EDGL-FLP [45] also achieves performance improvements through an effective strategy that prioritizes feature fusion. Due to the limited size of the training set in SFEW dataset, similar to other SOTA methods, we initially pre-train our LDR-Net on the RAF-DB dataset and subsequently fine-tune it on the SFEW dataset. Our LDR-Net achieves an accuracy of 62.65%, surpassing these SOTA results.

### 4.5. FER with Occlusion and Variant Pose in the Wild

To evaluate the performance of the LDR-Net under occlusion and pose variations, we conduct several evaluations on occlusion-aware datasets with the default setting, which include Occlusion RAF-DB, Occlusion-AffectNet, and FED-RO [3]), and pose-aware datasets including Pose-RAF-DB (Pose ≥ 30°), Pose-RAF-DB (Pose ≥ 45°), Pose-AffectNet (Pose ≥ 30°), and Pose-AffectNet (Pose ≥ 45°). The comparisons of our LDR-Net with several current SOTA methods, such as RAN [1], MA-Net [7], VTFF [30], AMP-Net [47], MRAN [33], and GLMEA [8], are shown in Table 7 and Table 8.

According to the experimental results in a in Table 7, LDR-Net achieves 89.80%, 92.70%, and 92.65% on Occlusion-RAF-DB, Pose-RAF-DB (Pose ≥ 30°), and Pose-RAF-DB (Pose ≥ 45°), respectively, outperforming all compared methods. Compared to the previous best method, MRAN [33], LDR-Net improves by 2.86%, 2.32%, and 2.87%, respectively. Notably, LDR-Net also surpasses the IR-50 baseline by 2.04%, 1.52%, and 1.61% on these three test sets. These improvements can be attributed to the proposed landmark-guided patch cropping strategy, which preserves spatial structure under occlusion and pose variations, and the dual-stream learning mechanism that captures both local dependencies and global context.

The results of the comparison on the AffectNet-7 dataset are shown in b in Table 7, which indicate that the proposed method achieves the highest accuracy, surpassing MRAN [33] by 3.52%, 2.96%, and 1.28% on Occlusion-AffectNet, Pose-AffectNet (Pose ≥ 30°), and Pose-AffectNet (Pose ≥ 45°), respectively. Additionally, as shown in c in Table 7, on Occlusion-AffectNet-8, LDR-Net achieves 64.28%, surpassing the IR-50 baseline by 1.62%. For Pose-AffectNet-8, LDR-Net improves over the baseline by 2.31% (Pose ≥ 30°) and 1.93% (Pose ≥ 45°), with accuracies of 61.57% and 61.32%, respectively.

**Figure 6:**
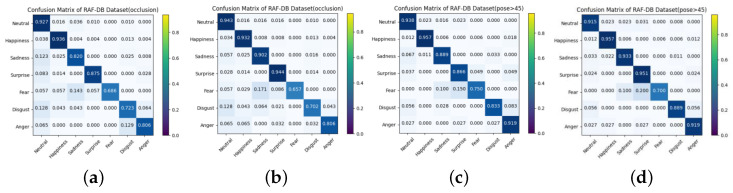
Confusion matrices on the Occlusion-RAF-DB and Pose-RAF-DB test sets. (**a**) Baseline on Occlusion-RAF-DB; (**b**) LDR-Net on Occlusion-RAF-DB; (**c**) Baseline on Pose-RAF-DB (Pose ≥ 45°); (**d**) LDR-Net on Pose-RAF-DB (Pose ≥ 45°).

**Figure 7:**
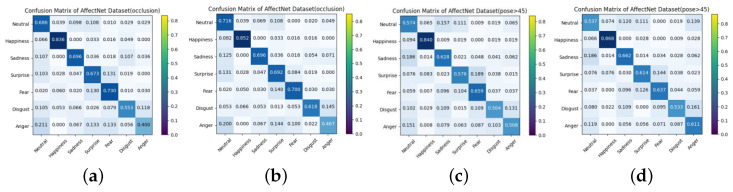
Confusion matrices on the Occlusion-AffectNet and Pose-AffectNet test sets. (**a**) Baseline on Occlusion-AffectNet-7; (**b**) LDR-Net on Occlusion-AffectNet-7; (**c**) Baseline on Pose-AffectNet-7 (Pose ≥ 45°); (**d**) LDR-Net on Pose-AffectNet-7 (Pose ≥ 45°).

Furthermore, we present the confusion matrices of our LDR-Net and the baselines in Figure 6 and Figure 7. We find that our LDR-Net consistently boosts the *Sadness* and *Surprise* categories on all the test sets. This may be attributed to our LRR’s ability to effectively capture distinct local patch features, such as action units associated with “Cheek Raiser” and “Lip Corner Depressor”. Furthermore, we observe notable performance improvements in the *Disgust* and *Anger* categories within the AffectNet dataset. These findings indicate that the diverse regional feature design of LDR-Net effectively reduces inter-class similarities, improving classification accuracy.

In addition, we conduct a fair comparison on the recent occlusion test dataset: FED-RO [3]. Following the standard cross-dataset protocol as [3], we train LDR-Net on the combined training set of RAF-DB and AffectNet, and evaluate it on the independent FED-RO. Table 8 presents the experimental results, where our method achieves state-of the-art performance with an accuracy of 73.75%. When compared to RAN [1], MA-Net [7], AMP-Net [47], and EDGL-FLP [45], our method leads by 5.77%, 3.75%, 2.00%, and 1.50%, respectively. Even compared to GLMEA [8] (72.59%), which recently achieved the highest accuracy, our approach still maintains a 1.16% lead, demonstrating LDR-Net’s excellent robustness to both occlusion and pose variations.

### 4.6. Experiment Results of LDR-Net for FER in Driving Environment

**Figure 8:**
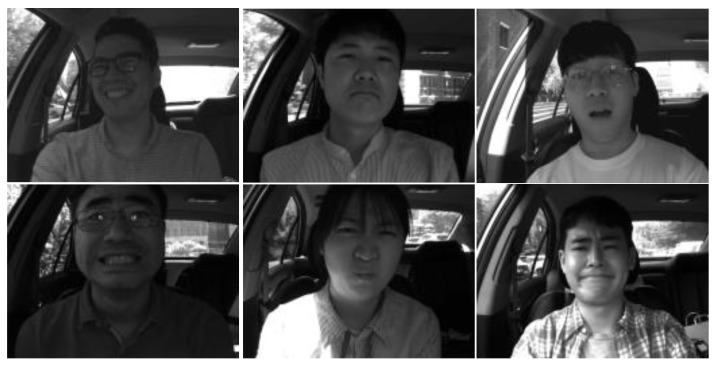
Examples from the KMU-FED dataset, highlighting the unique challenges of FER in driving environments, such as lighting variations and limited user cooperation, compared to conventional FER.

To further evaluate the generalization capability of LDR-Net in real-world driving environments and address the potential issue of subject leakage, we conduct experiments on KMU-FED under two different validation protocols. Representative samples from this dataset are shown in Figure 8.

To facilitate comparison with previous studies, we first report results under a random split-based cross-validation protocol, which is broadly consistent with the evaluation settings used in prior works. As shown in Table 9, LDR-Net achieves an accuracy of 99.10%, outperforming several representative methods.

However, random cross-validation may suffer from subject overlap between training and test sets, potentially leading to subject leakage and overly optimistic performance estimates. To rigorously evaluate generalization to unseen subjects, we further conduct leave-one-subject-out (LOSO) validation.

As reported in Table 10, LDR-Net achieves an average accuracy of 86.24±10.54% across 12 subjects. The relatively large standard deviation mainly stems from the inherent class imbalance in KMU-FED: due to its limited size, the validation set for certain subjects lacks samples from several categories, leading to missing evaluation coverage and increased confusion among visually similar expressions. Thus, the performance fluctuation largely reflects dataset-level incompleteness rather than model instability. Nevertheless, the LOSO result demonstrates that LDR-Net maintains competitive recognition performance on unseen subjects, indicating its potential for practical FER in driving environments.

### 4.7. Computational Efficiency Analysis

To assess the practical deployment potential of LDR-Net, we evaluate its computational efficiency in terms of model parameters (Params), floating-point operations (FLOPs), inference speed (FPS), and peak GPU memory usage. All measurements are conducted on an NVIDIA RTX 4090 GPU with an input resolution of 224×224. Model parameters and FLOPs are computed using the thop library, while FPS is averaged over 100 forward passes after 10 warm-up iterations.

As reported in Table 11, LDR-Net achieves an inference speed of 413.9 FPS with a batch size of 64, while requiring a peak GPU memory footprint of 3839.4 MB. These results demonstrate that LDR-Net maintains a favorable balance between recognition accuracy and computational cost.

To provide broader context, we also examined the computational metrics reported in the original publications of the KMU-FED baseline methods. Hierarchical WRF [18] and LMRF [48] report efficiency on CK+ (0.25 M/0.0067 M and 1.32 M/0.036 M FLOPs), while ILAB-CNN [49] reports 1.29 M parameters and 2.44 ms inference time on RAF-DB. Under our evaluation protocol on RTX 4090, LDR-Net achieves 2.42 ms per sample with 413.9 FPS (BS = 64). Despite differences in hardware and datasets, both methods exhibit comparable inference speed, yet LDR-Net achieves substantially higher accuracy (92.11% vs. 85.03% on RAF-DB). This suggests that LDR-Net maintains competitive efficiency while delivering significantly better performance, though the comparison serves as a practical reference rather than a direct one.

It is worth noting that the total parameter count of LDR-Net (88.47 M) includes the pre-trained facial landmark detector (SLPT) and the IR50 backbone (56.92 M in total), which are adopted for fair comparison with prior works. The proposed core modules (DRFE, DRL, and HFF) collectively account for 31.55 M parameters, representing 35.7% of the total. This indicates that the majority of parameters come from pre-trained components, and the overall model size can be effectively reduced by replacing these components with more compact alternatives for resource-constrained scenarios.

### 4.8. Visualization of LRR in DRL

**Figure 9:**
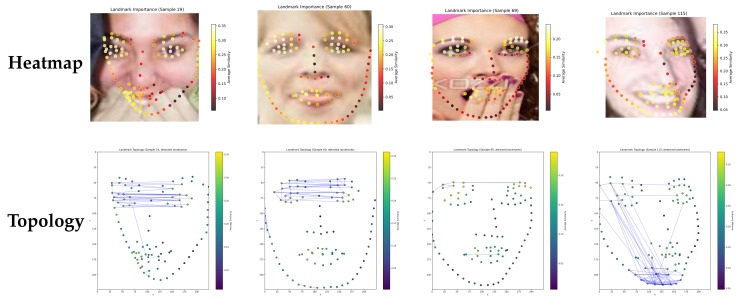
Visualization of the LRR on four representative samples from the RAF-DB test set. (**Top row**): landmark heatmaps based on average similarity to other landmarks. (**Bottom row**): corresponding landmark topologies showing pairwise similarities above 0.5. The visualization is based on 128 randomly selected samples, from which four are shown for illustration.

To provide interpretability for the LRR in DRL, we visualize four representative samples from RAF-DB test set in Figure 9. The top row shows landmark heatmaps overlaid on the input faces, where brighter colors indicate higher average similarity with other landmarks in LRR. The bottom row presents the corresponding landmark topologies, where connecting lines indicate strong pairwise similarities (thresholded at 0.5). The resulting topological structures, characterized by dense intra-regional connections and sparse inter-regional links, reflect the coordinated activation patterns of facial action units, enabling the model to capture fine-grained and structurally meaningful representations for accurate expression recognition.

### 4.9. Limitations and Failure Cases

**Figure 10:**
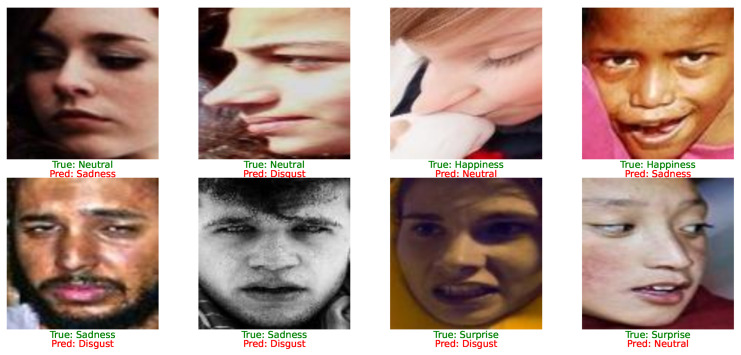
Representative failure cases of LDR-Net on the RAF-DB test set. Ground-truth and predicted labels are indicated in green and red, respectively.

To better understand the limitations of LDR-Net, Figure 10 presents eight representative misclassified samples from the RAF-DB test set. These examples illustrate several representative failure modes encountered by our LDR-Net under unconstrained FER.

Three representative error patterns can be observed. First, confusion between *sadness* and *disgust* occurs repeatedly, which is consistent with previous FER studies, as these two expressions share similar facial configurations and overlapping facial action units. Second, low-intensity positive expressions, including *happiness* and *surprise*, are occasionally misclassified as *neutral*, suggesting that subtle facial movements remain difficult to distinguish from neutral appearances. Third, several *neutral* samples are incorrectly classified as negative emotions such as *sadness* or *disgust*, likely due to subtle facial texture variations or ambiguous facial cues that lead the model to overestimate negative affect. These failure cases suggest that distinguishing subtle expression intensities and highly similar expression categories remains challenging, even with the proposed global–local collaborative architecture.

## 5. Conclusions

In this paper, we propose LDR-Net for facial expression recognition (FER) in the wild. Unlike existing methods that overlook spatial structure when seeking diverse region-specific features due to fixed or limited local regions, LDR-Net leverages facial landmarks as dynamic patch centers to locate expression-critical areas while preserving structural details. The framework consists of three synergistic modules: DRFE extracts region-specific features via landmark-guided cropping and cross-level integration with feature reorganization; DRL captures fine-grained local dependencies through Transformer-based LRR cells while reinforcing global features via CNN-based GRR cells; and HFF performs joint decision optimization via hierarchical hybrid fusion. The effectiveness of LDR-Net can be attributed to its bio-inspired design: facial landmarks serve as dynamic spatial anchors for structure-preserving region localization, the dual-stream DRL module captures both fine-grained local dependencies and global context in parallel, and the HFF module enables adaptive fusion of complementary representations. This integrated multi-granularity strategy yields more discriminative features and accounts for the observed performance gains. Extensive experiments on three FER benchmarks and five occlusion/pose test sets demonstrate that LDR-Net achieves state-of-the-art performance across various challenging scenarios, confirming its practical applicability for real-world deployment.

Future work will focus on two directions: (1) developing more efficient landmark-guided attention mechanisms and lightweight architectures for real-time deployment; and (2) extending the framework to other fine-grained facial analysis tasks such as facial action unit detection and pain estimation; (3) evaluating cross-dataset generalization (e.g., RAF-DB ↔ AffectNet) and robustness to landmark localization uncertainties (e.g., noisy, shifted, and missing landmarks).

## Figures and Tables

**Table 1:** Expression category distribution for each dataset used in our experiments.

Dataset	Year	Facial Expression Categories								Total
		Neutral	Happy	Surprise	Sad	Angry	Disgust	Fear	Contempt	
RAF-DB [2]	2017	3204	5957	1619	2460	867	877	355	-	15,339
AffectNet [38]	2017	75,374	134,915	14,590	25,959	25,383	4303	6878	4251	291,653
SFEW [39]	2011	229	257	151	235	256	76	125	-	1329
FED-RO [3]	2019	50	59	63	66	53	51	58	-	400
KMU-FED [18]	2018	-	210	200	180	196	120	200	-	1106

**Table 2:** Evaluation (%) of each component in LDR-Net on RAF-DB dataset.

Settings	Models	Acc	Mean Acc
a	Baseline ^‡^	90.87	84.73
b	+DRFE_Gs	91.59	85.29
c	+DRFE_Gs + GRR	91.82	85.56
d	+DRFE	91.88	85.18
e	+DRFE + DRL	91.92	86.14
f	+LDR-Net ^‡^	**92.11**	**86.55**

^‡^ Mean ± std over five runs. Baseline: 90.80±0.05%, LDR-Net: 91.98±0.08%, p<0.01.

**Table 3:** Evaluation (%) of different fusion strategies on RAF-DB dataset.

Fusion Strategies	Acc (%)	Mean Acc (%)
Feature-level	91.66	85.16
Decision-level	91.85	85.33
Hybrid-level	**92.11**	**86.55**

**Table 4:** Comparison (%) with SOTA methods on RAF-DB dataset.

Methods	Year	Backbone	FLs	RAF-DB
RAN [1]	2020	ResNet-18	✓	86.90
SCN [20]	2020	ResNet-18	✓	87.03
MA-Net [7]	2021	ResNet-18	✗	88.42
FDRL [4]	2021	ResNet-18	✗	89.47
TransFER [6]	2021	IR-50	✗	90.91
SEIIL [42]	2022	ResNet-50	✓	88.23
Mata-Face2Exp [43]	2022	ResNet-50	✗	88.54
DAN [21]	2023	ResNet-18	✗	89.70
MGR^3^Net [23]	2023	ResNet-18	✗	88.82
MGR^3^Net [23]	2023	ResNet-50	✓	91.05
MM-Net [44]	2024	ResNet-50	✗	89.77
GLMEA [8]	2025	ResNet-18	✗	90.09
WGGLFA [25]	2025	ResNet-34	✓	90.32
LDR-Net (ours)		ResNet-18	✗	88.95
LDR-Net (ours)		IR-50	✓	**92.11**

✓ and ✗ indicate whether facial landmarks (FLs) are used.

**Table 5:** Comparison (%) with SOTA methods on AffectNet-7 and AffectNet-8 dataset.

Methods	Year	AffectNet-7	AffectNet-8
RAN [1]	2020	-	59.50
SCN [20]	2020	-	60.23
MA-Net [7]	2021	64.53	60.29
TransFER [6]	2021	66.23	-
EAC [46]	2022	65.32	-
Mata-Face2Exp [43]	2022	64.23	-
DAN [21]	2023	65.69	62.09
MGR^3^Net [23]	2023	66.36	-
EDGL-FLP [45]	2023	-	61.25
MM-Net [44]	2024	65.05	-
GLMEA [8]	2025	65.40	-
Baseline (IR50)	-	64.91	61.75
LDR-Net	-	**67.14**	**63.20**

**Table 6:** Comparison (%) with SOTA methods on SFEW dataset.

Methods	Year	Acc (%)
RAN [1]	2020	56.40
MA-Net [7]	2021	59.40
FDRL [4]	2021	62.16
DAN [21]	2023	57.88
EDGL-FLP [45]	2023	62.31
Baseline (IR50)	-	60.32
LDR-Net	-	**62.65**

**Table 7:** Comparison (%) with state-of-the-art methods on occlusion and pose-changes datasets.

(a) The results on Occlusion-RAF-DB and Pose-RAF-DB.				
**Methods**	**Year**	**Occlusion**	**Pose ≥ 30°**	**Pose ≥ 45°**
RAN [1]	2020	82.72	86.74	85.20
MA-Net [7]	2021	83.65	87.89	87.99
VTFF [30]	2021	83.95	87.97	88.35
AMP-Net [47]	2022	85.28	89.75	89.25
MRAN [33]	2023	86.94	90.38	89.78
MM-Net [44]	2024	84.90	89.74	89.07
GLMEA [8]	2025	86.36	89.81	89.77
Baseline (IR50)		87.76	91.18	91.04
LDR-Net		**89.80**	**92.70**	**92.65**
**(b) The results on Occlusion-AffectNet-7 and Pose-AffectNet-7.**				
**Methods**	**Year**	**Occlusion**	**Pose ≥ 30°**	**Pose ≥ 45°**
RAN [1]	2020	58.50	53.90	53.19
MA-Net [7]	2021	59.59	57.51	57.48
VTFF [30]	2021	62.98	60.61	61.00
AMP-Net [47]	2022	64.27	61.37	61.16
MRAN [33]	2023	63.54	61.42	62.05
Baseline (IR50)		64.70	61.81	60.74
LDR-Net		**67.06**	**64.38**	**63.33**
**(c) The results on Occlusion-AffectNet-8 and Pose-AffectNet-8.**				
**Methods**	**Year**	**Occlusion**	**Pose ≥ 30°**	**Pose ≥ 45°**
Baseline (IR50)		62.66	59.26	59.39
LDR-Net		**64.28**	**61.57**	**61.32**

**Table 8:** Evaluation (%) on the FED-RO dataset.

Methods	Year	Acc (%)
RAN [1]	2020	67.98
MA-Net [7]	2021	70.00
AMP-Net [47]	2022	71.75
EDGL-FLP [45]	2023	72.25
MGR^3^Net [23]	2023	71.38
MM-Net [44]	2024	68.75
WGGLFA [25]	2025	71.90
GLMEA [8]	2025	72.59
LDR-Net (ours)	-	**73.75**

**Table 9:** Performance comparison on the KMU-FED dataset using random 10-fold cross-validation.

Methods	Year	Acc (%)
Hierarchical WRF [18]	2018	94.70
LMRF [48]	2020	95.10
ILAB-CNN [49]	2025	98.80
LDR-Net (ours)	-	**99.10**

**Table 10:** Subject-independent evaluation of LDR-Net on KMU-FED using leave-one-subject-out (LOSO) validation.

Protocol	Acc (%)	Range (%)
LOSO (12 subjects)	**86.24** ± **10.54**	[68.57, 100]

**Table 11:** Computational efficiency of the proposed LDR-Net.

Metric	Value
Parameters (M)	88.47
FLOPs (G)	13.83
Inference Speed (FPS @ batch size = 64)	413.9
Peak GPU Memory (MB)	3839.4

## Data Availability

The facial expression recognition datasets used in this study (RAF-DB, AffectNet, SFEW, and KMU-FED) are publicly available. No new datasets were generated. The source code for the proposed LDR-Net is not publicly available due to pending patent applications but is available from the corresponding author upon reasonable request.
